# Aquatic habitats of the malaria vector *Anopheles funestus* in rural south-eastern Tanzania

**DOI:** 10.1186/s12936-020-03295-5

**Published:** 2020-06-23

**Authors:** Ismail H. Nambunga, Halfan S. Ngowo, Salum A. Mapua, Emmanuel E. Hape, Betwel J. Msugupakulya, Dickson S. Msaky, Nicolaus T. Mhumbira, Karim R. Mchwembo, Gerald Z. Tamayamali, Slyakus V. Mlembe, Rukiyah M. Njalambaha, Dickson W. Lwetoijera, Marceline F. Finda, Nicodem J. Govella, Damaris Matoke-Muhia, Emmanuel W. Kaindoa, Fredros O. Okumu

**Affiliations:** 1 Environmental Health and Ecological Sciences Department, Ifakara Health Institute, P.O. Box 53, Ifakara, Tanzania; 2 School of Public Health, Faculty of Health Sciences, University of the Witwatersrand, Park Town, Republic of South Africa; 3 Institute of Biodiversity, Animal Health and Comparative Medicine, University of Glasgow, Glasgow, UK; 4 School of Life Science and Bioengineering, Nelson Mandela African Institution of Science & Technology, Arusha, Tanzania; 5 Centre for Applied Entomology and Parasitology, School of Life Sciences, Keele University, Newcastle-under-Lyme, UK; 6 Center for Biotechnology Research and Development, Kenya Medical Research Institute, Nairobi, Kenya

**Keywords:** *Anopheles funestus*, Ifakara, Malaria, Tanzania, Larviciding, Larval source management

## Abstract

**Background:**

In rural south-eastern Tanzania, *Anopheles funestus* is a major malaria vector, and has been implicated in nearly 90% of all infective bites. Unfortunately, little is known about the natural ecological requirements and survival strategies of this mosquito species.

**Methods:**

Potential mosquito aquatic habitats were systematically searched along 1000 m transects from the centres of six villages in south-eastern Tanzania. All water bodies were geo-referenced, characterized and examined for presence of *Anopheles* larvae using standard 350 mLs dippers or 10 L buckets. Larvae were collected for rearing, and the emergent adults identified to confirm habitats containing *An. funestus*.

**Results:**

One hundred and eleven habitats were identified and assessed from the first five villages (all < 300 m altitude). Of these, 36 (32.4%) had *An. funestus* co-occurring with other mosquito species. Another 47 (42.3%) had other *Anopheles* species and/or culicines, but not *An. funestus*, and 28 (25.2%) had no mosquitoes. There were three main habitat types occupied by *An. funestus,* namely: (a) small spring-fed pools with well-defined perimeters (36.1%), (b) medium-sized natural ponds retaining water most of the year (16.7%), and (c) slow-moving waters along river tributaries (47.2%). The habitats generally had clear waters with emergent surface vegetation, depths > 0.5 m and distances < 100 m from human dwellings. They were permanent or semi-permanent, retaining water most of the year. Water temperatures ranged from 25.2 to 28.8 °C, pH from 6.5 to 6.7, turbidity from 26.6 to 54.8 NTU and total dissolved solids from 60.5 to 80.3 mg/L. In the sixth village (altitude > 400 m), very high densities of *An. funestus* were found along rivers with slow-moving clear waters and emergent vegetation.

**Conclusion:**

This study has documented the diversity and key characteristics of aquatic habitats of *An. funestus* across villages in south-eastern Tanzania, and will form an important basis for further studies to improve malaria control. The observations suggest that *An. funestus* habitats in the area can indeed be described as fixed, few and findable based on their unique characteristics. Future studies should investigate the potential of targeting these habitats with larviciding or larval source management to complement malaria control efforts in areas dominated by this vector species.

## Background

*Anopheles funestus* has been a major malaria vector in many east and southern African countries for several years [1–4]. In south-eastern Tanzania, they have been implicated in more than 85% of malaria transmission events across several villages [4–6]. Its dominance in pathogen transmission [4, 7] is attributable to factors such as: (a) being predominantly anthropophilic (i.e. strong preference for blood from humans over other vertebrates) and endophilic (i.e. strong preference for biting and resting indoors than outdoors) [8, 9], (b) their resistance to some of the commonly-used pyrethroid insecticides in locations such as south-eastern Tanzania [10–14], and (c) their superior daily survival probabilities as reflected in the higher parity rates compared to other *Anopheles* species [4, 5, 7].

The supremacy of *An. funestus* in malaria transmission has been observed even in areas where they occur at far lower densities compared to other malaria vectors, such as *Anopheles arabiensis* [4, 5, 15]. In such settings, the infrequent occurrence partly explains why their behaviours are relatively understudied in the field. More generally, *An. funestus* is also far easier to find as adults than as larvae. As a result, this species rarely features in larval surveys of *Anopheles* species. Researchers, therefore, sometimes rely on adult collections rather than larval collections to obtain enough samples for insecticide resistance testing [4], which according to the World Health Organization (WHO) protocols require F1 offspring with synchronized age groups [16].

It has previously been suggested that an in-depth ecological understanding, followed by improved targeting of *An. funestus* could potentially improve their control, and significantly reduce malaria transmission in areas where the vector dominates [4]. Given the strong resistance of some *An. funestus* populations to insecticides commonly applied on insecticide-treated nets (ITNs) and/or indoor residual spraying (IRS) [10–14], supplementary measures targeting the aquatic stages of the mosquitoes are critical for more effective control of *An. funestus*. This requires rigorous surveys to identify and characterize preferred larval habitats for *An. funestus* [17]. Strategies such as targeted larviciding—a component of larval source management could indeed significantly improve control efforts and accelerate progress towards malaria elimination, especially in communities where the aquatic habitats are fixed, few and findable [18, 19].

A previous study in western Kenya reported that *An. funestus* prefers to oviposit in large semi-permanent water bodies containing aquatic vegetation and algae [20]. A separate study in coastal Kenya observed these species breeding in vegetated aquatic habitats that are stable and permanent, and were along river streams [21]. In Cameroon, it was demonstrated that *An. funestus* habitats were often found in open savannas instead of deep or degraded forests [22, 23]. These habitats had greater exposure to sunlight and high temperatures, and remained productive for longer, often with peaks after the start of the dry season. Unfortunately, in south-eastern Tanzania where the species now dominates transmission, there have not been detailed studies of its natural aquatic habitats and responses to interventions. This situation is complicated by difficulties in colonizing the species inside laboratories, which would enable such studies.

This current baseline study was, therefore, aimed at identifying and characterizing the main larval habitats of *An. funestus* to advance knowledge of its aquatic ecology. The findings were expected to provide a basis for further investigations into improved control strategies targeting the species, and also to inform ongoing efforts for rearing this species under laboratory conditions.

## Methods

### Study areas

**Fig. 1:**
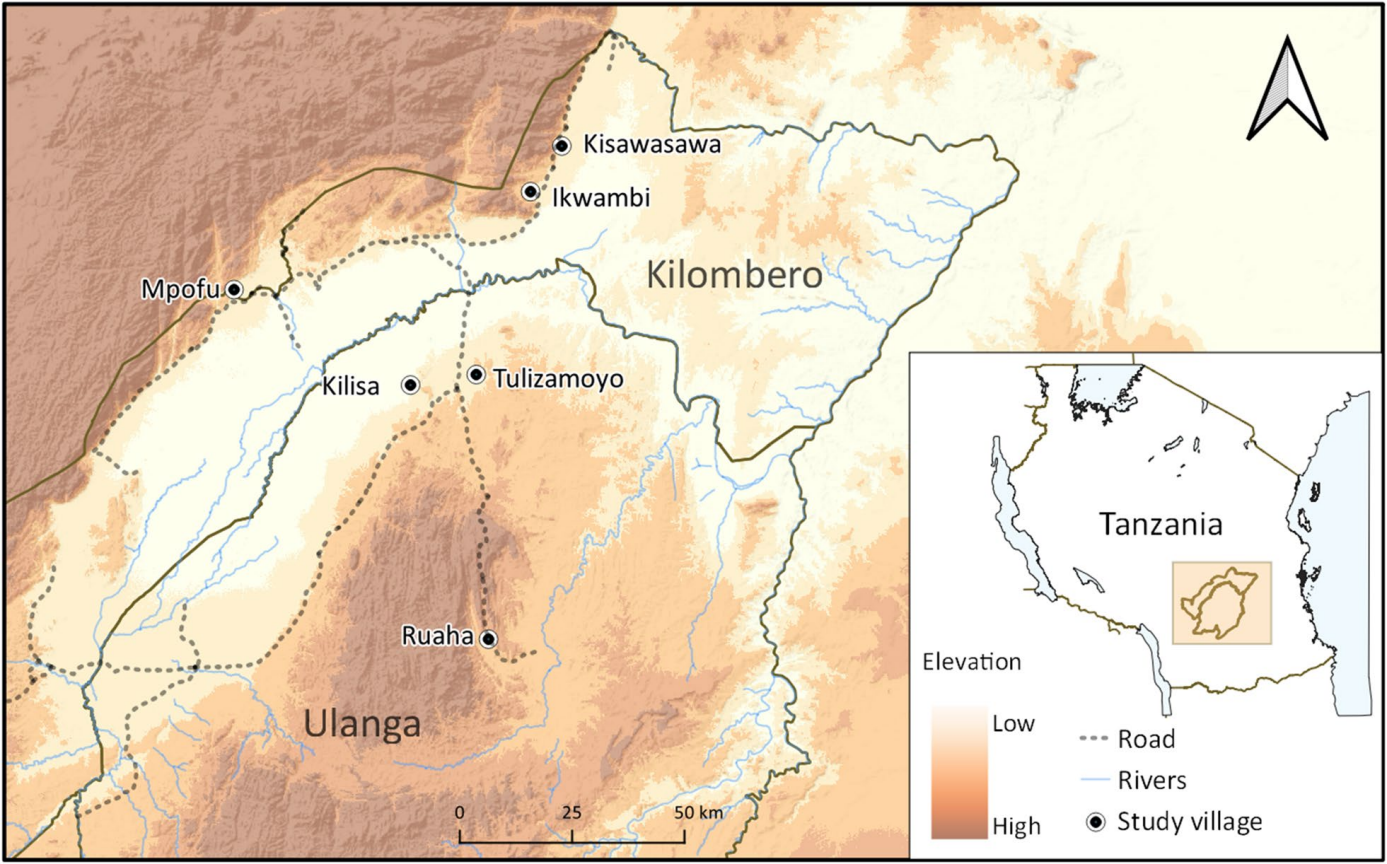
Map of Kilombero and Ulanga districts showing the six study villages

This study was conducted in six villages of Kilombero and Ulanga districts in south-eastern Tanzania (Fig. 1). Five of these villages were located at altitudes less than 300 m above sea level, while the sixth was at an altitude greater than 400 m. In Kilombero district, the study villages were Ikwambi (− 7.97927° S, 36.81630° E), Kisawasawa (− 7.89657° S, 36.88058° E) and Mpofu (− 8.17220° S, 36.21651° E), while in Ulanga district, the villages were Kilisa (− 8.37544° S, 36.57355° E), Ruaha (− 8.9063° S, 36.7194° E) and Tulizamoyo (− 8.35447° S, 36.70546° E). The study villages were selected based on the high abundance of adult *An. funestus* mosquitoes based on previous surveillance work done by Ifakara Health Institute (unpublished data). The annual rainfall and temperature ranges in these villages were 1200–1800 mm and 20–32.6 °C respectively. The main economic activities are crop farming (mostly rice and maize farming) and livestock keeping.

### Larvae collection and rearing

**Fig. 2:**
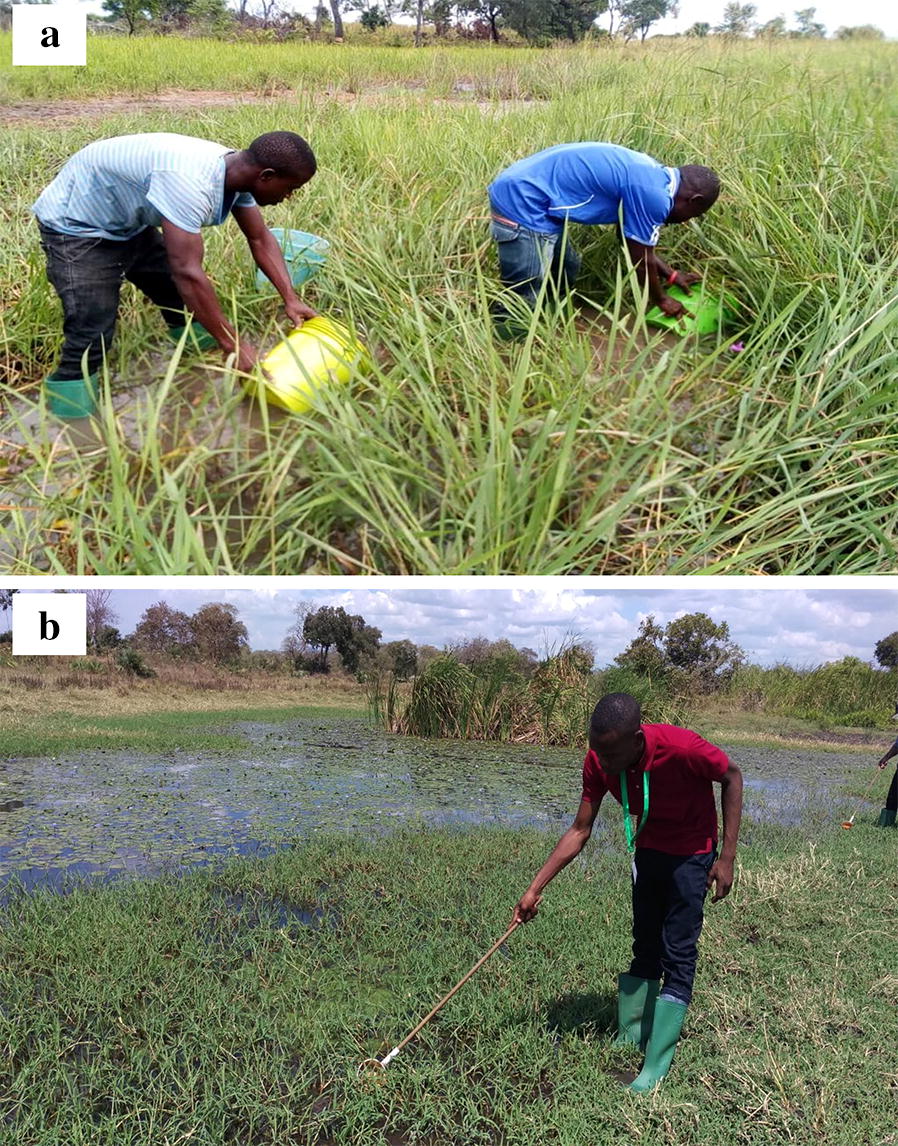
Collection of *An. funestus* larvae using 10 L bucket (**a**) and standard dipper (**b**)

This study was done between January and September 2018, and repeated between October and December 2019. The study villages were surveyed for the presence of aquatic habitats along transects of 1000 m, each radiating from an approximated village centroid. All identified water bodies were marked, geo-referenced, physically characterized and examined for the presence of *Anopheles* larvae. Standard 350 mL dippers or 10 L plastic buckets were used to sample water from the pools (Fig. 2). When the water bodies consisted of rivers and streams, larval sampling was done along the river length over distances not exceeding 1000 m, so as to match the 1000 m transects in the main survey. Parts of the rivers with or without *Anopheles* larvae were similarly characterized and geo-referenced. The buckets were used in sites where it was impractical to use the dippers (e.g. habitats with depths greater than 50 cm), and also to collect the larvae for further rearing and identification. The larvae collected from different aquatic sites were transported to the insectary at Ifakara Health Institute for rearing to adults.

Once in the insectary, the larvae were kept in rearing pans (32 cm diameter and 5 L holding capacity) labelled with information on the dates and place of larvae collection. The temperature in the insectary was kept at 26 °C ± 2 °C and relative humidity at 82% ± 10%. The larvae were fed with Tetramin^®^ fish food until they developed into pupae and emerged into adult mosquitoes. Emerging adult mosquitoes were collected using mouth aspirator, killed by freezing and all *Anopheles* were identified using morphology-based identification keys developed by Gilles and Coetzee [9, 24]. All identified *An. funestus* mosquitoes were then packed individually in 1.5 mL Eppendorf tubes with silica gel and submitted to molecular laboratory for sibling species identification by polymerase chain reaction (PCR) assays as described by Koekemoer et al. [25]. Habitats positive for *An. funestus* were then identified among all the surveyed habitats.

### Characteristics of aquatic habitats

Characteristics of all the aquatic habitats as well as the surrounding environments were recorded. For the habitats, information collected included water movement (stagnant or slow), water colour (clear, coloured, or polluted), a tree canopy (shade) over habitat (none, partial, heavy), habitat size in circumference (less than 10 m, between 10 and 100 m, more than 100 m), vegetation type (none, submerged, floating, emergent), vegetation quantity (none, scarce, moderate, abundant), algae quantity (none, scarce, moderate), water depth (less than 10 cm, between 10 and 50 cm, more than 50 cm), distance from the nearest homes (less than 100 m, between 100 and 500 m, more than 500 m) and water type (semi-permanent, permanent). The habitats were considered temporary, semi-permanent or permanent if retained water for less than 3 months, 3–9 months and throughout the year respectively.

Additionally, the physicochemical characteristics of water in the larval habitats were assessed in four of the six villages, namely Tulizamoyo, Ikwambi, Kisawasawa and Kilisa. Parameters assessed included: water temperature (°C), pH (scale of 0–14), conductivity (Siemens/m), total dissolved solids (mg/L) and turbidity (nephelometric turbidity units, using 2100Q portable turbidity meter). Assessments of these parameters were conducted in the field sites immediately after the collection of larvae from the habitats. Lastly, nitrate levels (milligrams per litre) were also analysed by spectrophotometric method. To do this, one litre of water samples from each habitat in the study sites was collected, stored in a cooler box and sent to the laboratory at Ifakara Health Institute for analysis within 24 h post collection.

### Data analysis

Analysis was done using open source software, R programming language [26]. A total of 16 environmental variables were used to identify the main predictors for the presence of *An. funestus* larvae in the study villages. At first, all main predictors were initially assessed individually using univariate logistic regression and assess its impact on the presence of *An. funestus* larvae. Secondly, all the variables were included in the final model and assess their effect on the presence of *An. funestus* larvae. Odds ratios and their 95% confidence intervals are reported, and the statistical differences were considered significant when P-values < 0.05.

## Results

### Occurrence of *An. funestus* and other mosquito species in different habitats

A total of 111 potential habitats were surveyed of which 83 (74.8%) were identified to have larvae while 28 (25.2%) did not have. Of the 83 larval habitats that were positive for mosquito larvae, 36 (43.4%) had *An. funestus*. More than two-thirds of the *An. funestus* habitats (69.4%; n = 25) were shared with *Culex* mosquitoes, while one third (30.6%; n = 11) were shared with other *Anopheles* species. The *An. funestus* habitats included: spring-fed pools (36.1%; n = 13), medium-sized natural ponds (16.7%; n = 6) and river tributaries with slow-moving waters (44.2%; n = 17).

Adult mosquitoes that emerged from the different sampled habitats consisted of: *An. funestus* sensu lato (s.l.) (64%; n = 696), *Culex* spp. (24.5%; n = 267)*, Anopheles coustani* (6.2%; n = 67)*, Anopheles gambiae* s.l. (4.3%; n = 47) and other species (1%; n = 11). PCR identification of the 501 *An. funestus* group revealed that 53.3% (n = 267) were *An. funestus* sensu stricto (s.s.), 28.7% (n = 144) were *Anopheles rivulorum*, 11.8% (n = 59) were *Anopheles leesoni* and 6.2% (n = 31) were unidentified due to non-amplification in the PCR assays. The *An. funestus* s.s. commonly shared habitats with the other sibling species including *An. leesoni* and *An. rivulorum*.

### Habitat characteristics

Table 1 summarizes different environmental variables in aquatic habitats associated with the presence of *An. funestus* and other mosquito species. These variables were assessed individually and later combined in the final model to see how they influence the presence of *An. funestus* larvae. Results from univariate logistic regression showed that, the permanent habitats with emergent vegetation were strongly associated with the presence of *An. funestus* larvae (P < 0.01). The final model, multivariate outputs show that stagnant or slow-moving water did not significantly affect the presence of *An. funestus* larvae from the observed aquatic habitats (Table 2). However, heavily shaded aquatic habitats (with high densities of tree canopy), especially along the rivers were more likely to harbour *An. funestus* larvae compared to others (Table 2). Furthermore, the aquatic habitats with a depth greater than 50 cm and vegetation were significantly associated with the presence of *An. funestus* larvae (Table 2).

**Table 1:** Characteristics of aquatic habitats of *An. funestus* and other mosquito species

Larval habitat	All water bodies n (%)	Water bodies without larvae n (%)	Habitats with *An. funestus* n (%)	Habitats with other *Anopheles* n (%)	Habitats with culicines n (%)
Water movement					
Stagnant	91 (82)	25 (89.3)	27 (75)	18 (75)	21 (91.3)
Slow	20 (18)	3 (10.7)	9 (25)	6 (25)	2 (8.7)
Habitat shade (density of tree canopy)					
None	60 (54.1)	11 (39.3)	17 (47.2)	18 (75)	14 (60.9)
Partial	35 (31.5)	14 (50)	11 (30.6)	4 (16.7)	6 (26.1)
Heavy	16 (14.4)	3 (10.7)	8 (22.2)	2 (8.3)	3 (13)
Water depth					
Less than 50 cm	51 (45.9)	12 (42.9)	12 (33.3)	17 (70.8)	10 (43.4)
Greater than 50 cm	60 (54.1)	16 (57.1)	24 (66.7)	7 (29.2)	13 (56.6)
Distance to human dwellings					
Less than 100 m	77 (69.4)	26 (92.9)	28 (77.8)	9 (37.5)	14 (60.9)
Greater than 100 m	34 (3.6)	2 (7.1)	8 (22.2)	15 (62.5)	9 (39.1)
Water type					
Permanent	44 (39.6)	7 (25)	21 (58.3)	7 (29.2)	9 (39.1)
Semi-permanent	67 (60.4)	21 (75)	15 (41.7)	17 (70.8)	14 (60.9)
Vegetation type					
Emergent	51 (46)	6 (21.4)	26 (72.2)	7 (29.2)	12 (52.2)
Submerged	11 (9.9)	7 (25)	1 (2.8)	1 (4.2)	2 (8.7)
None	25 (22.5)	12 (42.9)	3 (8.3)	6 (25)	4 (17.4)
Floating	24 (21.6)	3 (10.7)	6 (16.7)	10 (41.6)	5 (21.7)
Water colour					
Clear	59 (53.2)	7 (25)	29 (80.5)	15 (62.5)	8 (34.8)
Coloured	42 (37.8)	18 (64.3)	6 (16.7)	8 (33.3)	10 (43.5)
Polluted	10 (9)	3 (10.7)	1 (2.8)	1 (4.2)	5 (21.7)
Vegetation quantity					
None	24 (21.6)	11 (39.3)	3 (8.3)	6 (25)	4 (17.4)
Scarce	57 (51.4)	15 (53.5)	15 (41.7)	12 (50)	15 (65.2)
Moderate	22 (19.8)	1 (3.6)	13 (36.1)	5 (20.8)	3 (13.1)
Abundant	8 (7.2)	1 (3.6)	5 (13.9)	1 (4.2)	1 (4.2)
Algae quantity					
None	70 (63.1)	14 (50)	21 (58.3)	18 (75)	17 (73.9)
Scarce	32 (28.8)	11 (39.3)	13 (36.1)	4 (16.7)	4 (17.4)
Moderate	9 (8.1)	3 (10.7)	2 (5.6)	2 (8.3)	2 (8.7)
Habitat size					
Less than 10 m	61 (55)	22 (78.6)	13 (36.1)	12 (50)	14 (60.9)
Between 10 and 100 m	46 (41.4)	6 (21.4)	21 (58.3)	11 (45.8)	8 (34.8)
Greater than 100 m	4 (3.6)	0 (0)	2 (5.6)	1 (4.2)	1 (4.3)

**Table 2:** Results of univariate and multivariate regression analysis of different habitat characteristics and their association with presence of *An. funestus* larvae

Larval habitat	Univariate analysis		Multivariate analysis	
	Odds (95% LC, UC)	P-values	Odds (95% LC, UC)	P-values
Water movement				
Stagnant	1		1	
Slow	1.94 [0.72, 5.21]	0.189	3.71 [0.81, 17.00]	0.091
Habitat shading (density of tree canopy)				
None	1		1	
Partial	1.16 [0.47, 2.87]	0.750	0.84 [0.22, 3.24]	0.795
Heavy	2.53 [0.82, 7.83]	0.107	7.35 [1.04, 51.78]	< 0.05
Water depth				
Less than 50 cm	1		1	
Greater than 50 cm	2.17 [0.95, 4.96]	0.067	5.72 [1.40, 23.42]	< 0.05
Distance to human dwellings				
Less than 100 m	1		1	
Greater than 100 m	0.54 [0.21, 1.35]	0.186	0.43 [0.12, 1.49]	0.184
Water type				
Semi-permanent	1		1	
Permanent	3.16 [1.39, 7.23]	< 0.01	3.07 [0.86, 10.99]	0.085
Vegetation type				
None	1		1	
Submerged	0.73 [0.07, 7.95]	0.799	0.55 [0.21, 1.42]	0.216
Emergent	7.63 [2.03, 28.70]	< 0.01	1.96 [0.66, 5.78]	0.966
Floating	2.44 [0.53, 11.17]	0.249	0.92 [0.03, 31.86]	0.962
Water colour				
Clear	1		1	
Coloured	0.17 [0.06, 0.47]	< 0.001	0.10 [0.02, 0.46]	< 0.01
Polluted	0.11 [0.01, 0.97]	< 0.05	0.15 [0.01, 1.72]	0.127
Vegetation quantity				
None	1		1	
Scarce	2.50 [0.65, 9.60]	0.182	12.62 [1.76, 90.55]	< 0.05
Moderate	10.11 [2.31, 44.35]	< 0.01	20.03 [2.41, 166.2]	< 0.01
Abundant	11.67 [1.79, 76.01]	< 0.05	22.96 [1.33, 395.7]	< 0.05
Algae quantity				
None	1		1	
Scarce	1.60 [0.67, 3.82]	0.293	5.40 [1.35, 21.64]	< 0.05
Moderate	0.67 [0.13, 3.48]	0.631	0.50 [0.03, 7.97]	0.623
Habitat size				
Less than 10 m	1		1	
Between 10 and 100 m	3.10 [1.33, 7.21]	< 0.01	1.37 [0.37, 5.04]	0.638
Greater than 100 m	3.69 [0.47, 28.78]	0.212	1.17 [0.08, 17.07]	0.910

**Fig. 3:**
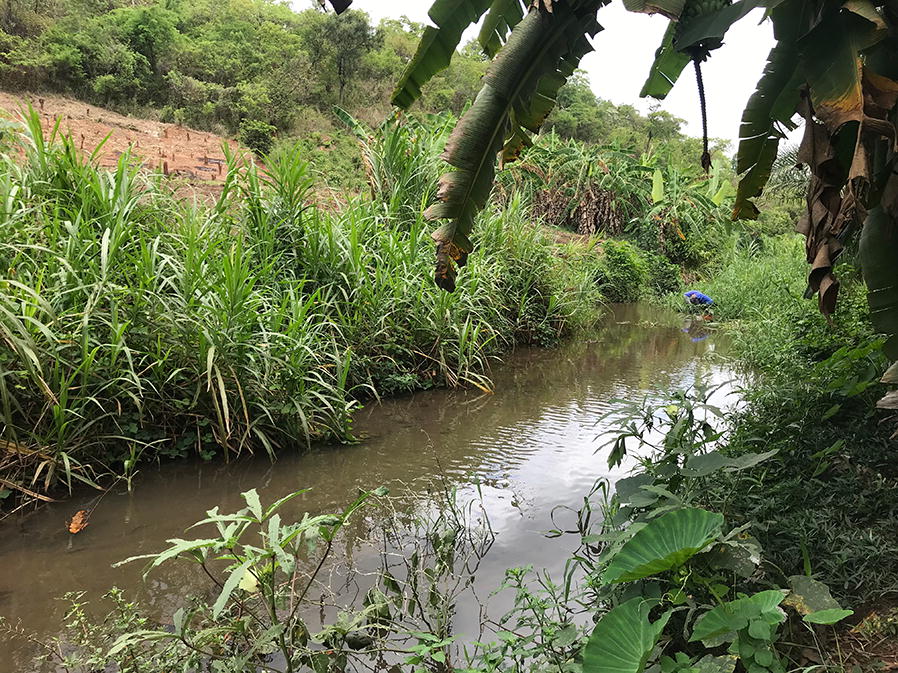
Picture of a riverside aquatic habitat for *Anopheles funestus* mosquitoes, as identified in the study areas in rural south-eastern Tanzania. At altitudes above 400 m, these were the only *An. funestus* habitats identified

**Fig. 4:**
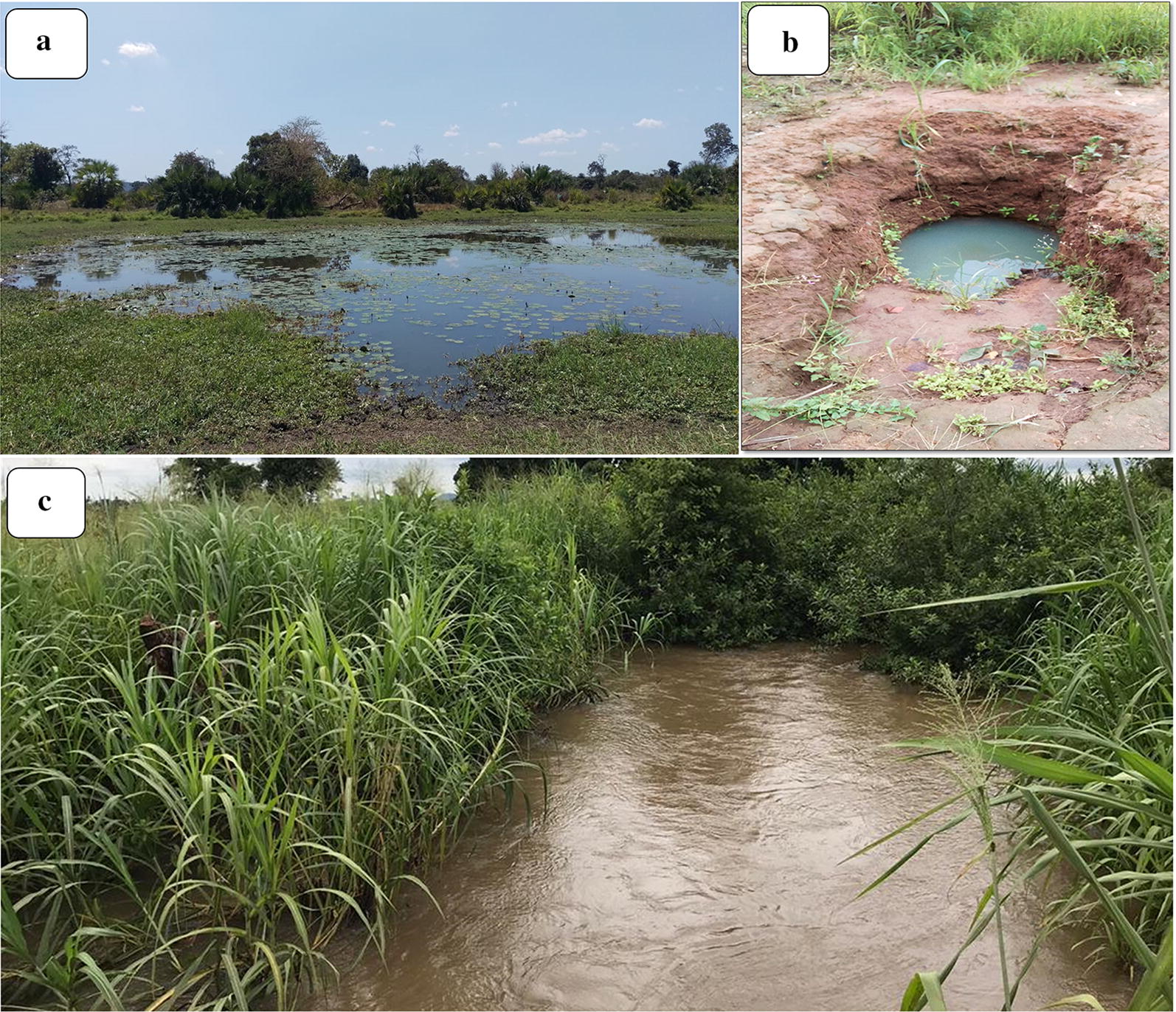
Typical larval habitats of *Anopheles funestus* mosquitoes in lower altitude areas (**a** medium-sized ponds that retain water at the centre most of the year and have emergent surface vegetation and **b** small spring-fed wells with well-defined perimeters) and habitats at higher altitudes (**c** slow-moving waters at the riverside with emergent vegetation)

**Fig. 5:**
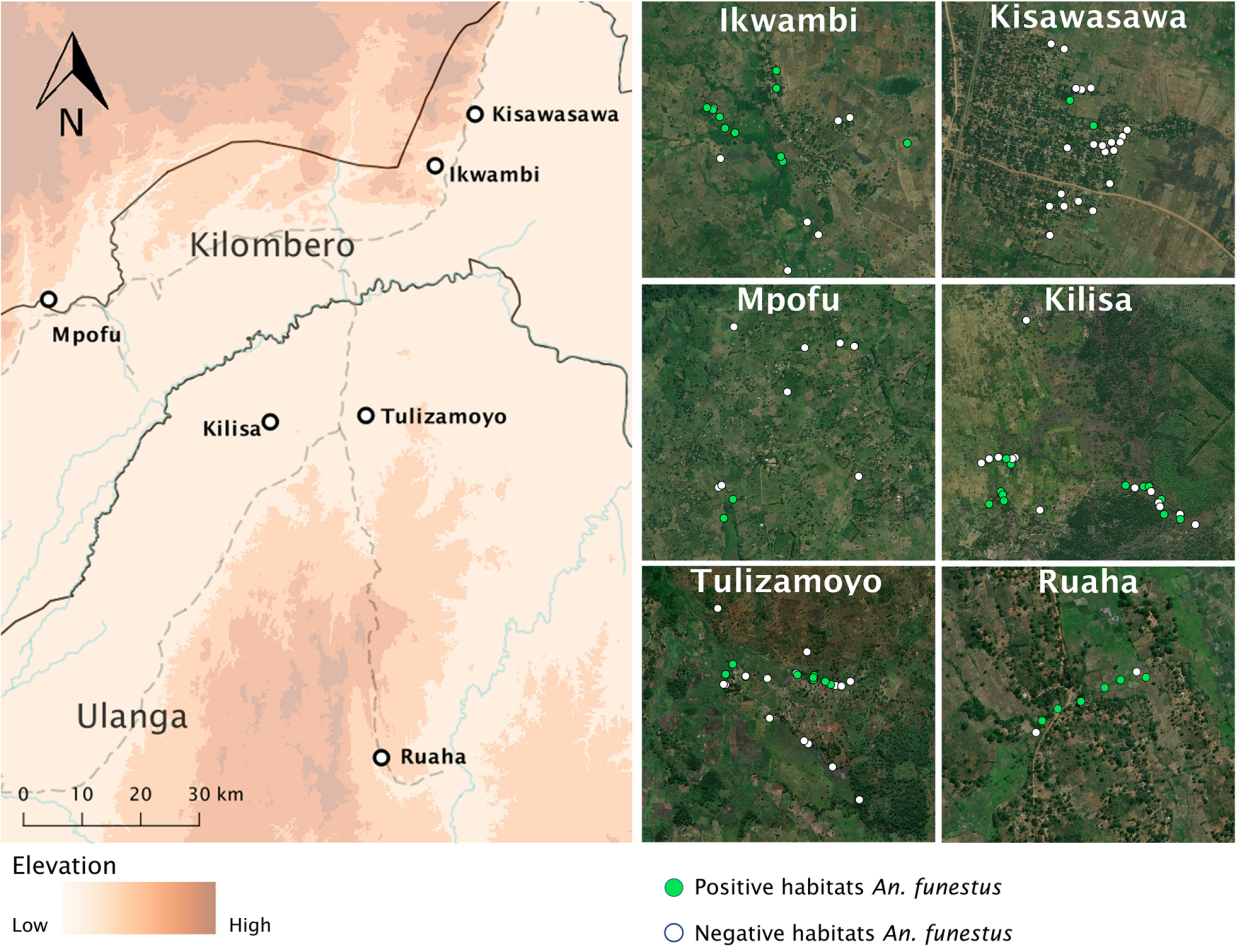
Spatial distribution of *Anopheles funestus* larval habitats in the selected study villages in south-eastern Tanzania

At higher altitudes, such as in Ruaha village, which was higher than 400 m above sea level, all the *An. funestus* larvae collected were from the rivers. The river sections acting as the breeding sites for *An. funestus* had slow-moving and clear waters near their banks. They were characterized by abundant emergent vegetation and water depths of greater than 50 cm (Fig. 3), and were within 100 m from human dwellings. The physical characteristics at these altitudes were the same as in the other habitats of *An. funestus* found below 300 m altitude, i.e. the natural perennial ponds, or small spring-fed water pools with well-defined areas (Figs. 4 and 5).

### Physicochemical characteristics of water in the aquatic habitats

Table 3 shows the median values of physicochemical parameters in larval habitats of different mosquito species. The pH in all *An. funestus* larval habitats were weakly acidic, ranging from 6.5 to 6.7. The concentration of total dissolved solids (tds) was highest in the water pools without any larvae (88.7–148.0 mg/L) and lowest in *An. funestus* habitats (60.5–80.3 mg/L). Turbidity was low in all habitats surveyed (11.5–64.0 NTU), while conductivity was higher in water pools without larvae (286.0 [99.2–310.0] µS/cm) compared to habitats containing *An. funestus* (151.0 [134.0–165.0] µS/cm) and others. The association between these physicochemical characteristics and the occurrence of *An. funestus* was however not statistically significant at P < 0.05 (Table 4).

**Table 3:** Median values of key physicochemical parameters in aquatic habitats dominated by different mosquito species

Dominant mosquito species	pH	Turbidity (NTU)	Temp (°C)	Conductivity (µS/cm)	TDS (mg/L)	Nitrate (mg/L)
*Anopheles funestus*	6.6 (6.5–6.7)	32.9 (26.6–54.8)	27.1 (25.2–28.8)	151.0 (134–165)	69.7 (60.5–80.3)	4.1 (2.9–6.6)
Culicine mosquitoes	6.2 (5.9–6.5)	36.0 (19.8–43.2)	26.5 (25.1–27.3)	161.0 (106–189)	78.8 (53.1–112.0)	2.7 (1.6–3.7)
Other *Anopheles*	6.8 (6.4–7.1)	24.9 (19.4–64.0)	28.9 (23.2–32.6)	211.0 (123–251)	102.0 (50.3–108.0)	10.3 (2.4–45.5)
Without larvae	6.41 (5.8–6.7)	15.6 (11.5–20.2)	25.6 (24.5–27.0)	286.0 (99.2–310.0)	142.0 (88.7–148.0)	2.45 (1.4–2.9)

**Table 4:** Univariate and multivariate analysis of associations between physicochemical parameters and the presence of *An. funestus* larvae

Characteristics	Univariate analysis		Multivariate analysis	
	Odds (95% LC, UC)	P-values	Odds (95% LC, UC)	P value
pH	1.40 [0.57, 3.44]	0.458	3.77 [0.96, 14.84]	0.057
Temperature	0.99 [0.86, 1.14]	0.896	0.92 [0.76, 1.12]	0.403
Nitrate	0.98 [0.94, 1.02]	0.368	0.94 [0.83, 1.01]	0.073
TDS	0.99 [0.98, 1.00]	0.197	0.98 [0.94, 1.02]	0.248
Turbidity	1.01 [0.99, 1.01]	0.285	1.01 [0.99, 1.02]	0.231
Conductivity	1.00 [0.99, 1.00]	0.272	1.01 [0.99, 1.02]	0.507

## Discussion

Although *An. funestus* are among the most important vectors of malaria in Africa, little is known regarding their larval ecology and development. This crucial gap needs an urgent solution, but is perpetuated by the inability of most mosquito biologists to create laboratory colonies of this vector species. Understanding the basic environmental parameters that influence mosquitoes breeding and oviposition can improve the planning, development and deployment of new interventions to control malaria transmission [27]. This study identified and characterized larval habitats of *An. funestus* in south-eastern Tanzanian villages of Ulanga and Kilombero districts, where this mosquito species has been implicated in most malaria-infective bites [4, 6].

The study examined more than 100 potential habitats across six villages and identified three main habitat types. First were small water wells with well-defined edges and were spring-fed, some of which were also used by locals as domestic water sources (Fig. 4b). These habitats were often occupied by multiple species of the *An. funestus* group, and in some cases, they were shaded by large trees. The second type of habitat was medium-sized ponds, for which the central part retained water for all or most of the year. These habitats often had surface vegetation (Fig. 4a) and were occupied by multiple other *Anopheles* species, such as *An. arabiensis*. Third was the riverside habitats consisting of the slow-moving waters on the rivers or river tributaries, also with vegetation (Fig. 4c). These habitats were mostly found at altitudes above 400 m above sea level, unlike the other two habitats which were more common at lower altitudes below 300 m (Fig. 5). In summary, *An. funestus* in this area appears to prefer permanent and semi-permanent aquatic habitats with stagnant or slow-moving waters, emergent vegetation e.g. algae on swamp surfaces, clear waters at depths exceeding 50 cm and nearness to human dwellings.

This study provides a basis for designing future surveys and control operations targeting malaria, especially in places such as south-eastern Tanzania where *An. funestus* and *An. arabiensis* play a major role in malaria transmission [5, 6, 28–30]. This study has suggested that permanent or semi-permanent habitats characterized by emergent vegetation play a major role in the ecology of *An. funestus*. The findings are concurrent with past evidence from earlier investigations in Kenya [20, 21, 31]. Although this current study did not assess the seasonality of *An. funestus* larvae densities in the different habitats, the observed preference of permanent and semi-permanent water bodies explains the known seasonality of its adult densities in the same study villages as observed in recent entomological surveys [4, 30]. The adult densities of *An. funestus* tend to peak after the rains just before the dry seasons begin, and are sustained by the large permanent water bodies [20]. Although no detailed studies have been done in this area targeting *An. funestus* aquatic habitats, early accounts by Gillies and DeMeillon [9], as well as limited surveys done nearly 50 years ago in the Ifakara area (which neighbours the current study site) already suggested an association between the late peaks in *An. funestus* densities and the large perennial habitats [32].

Although there was no clear statistical association, the *An. funestus* habitats had depths greater than 50 cm and were located within 100 m from the human dwellings. This is likely due to the anthropophagic nature of these mosquitoes [33], and further explains the importance of this species in malaria transmission in these areas. Other *Anopheles* species, such as *An. gambiae,* which breed in open sunlit stagnant water pools [9, 20, 34] are also highly anthropophagic and generally occur near human habitations [35]. The ability of *An. funestus* to breed in the river waters is not unique to Tanzania, but has also been demonstrated in other places such as coastal Kenya [9, 21], and may be due to the higher levels of aeration and dissolved oxygen in such waters. Additional investigations are therefore required to further examine these details. A similar ecological niche has been described for *Anopheles pseudopunctipennis* in South America, which was successfully controlled by clearing the river waters of the algal blooms [36]. While it is unclear whether clearing the identified habitats of emergent vegetation would be suitable for control of *An. funestus* in Tanzania, it will be important to investigate it as a potential environmentally-friendly approach, in which community members could be engaged to achieve effective disease prevention. Besides, it will be important to ascertain the importance of these habitat types across multiple sites and settings. For instance, in one area in the north of Tanzania, Dida et al. [37] found no mosquito larvae near the main rivers, suggesting the dominant malaria vectors may be breeding elsewhere in such settings.

Understanding the physicochemical characteristics of mosquito larval habitats is also important in understanding their overall ecological needs, and assessing options for manipulation. It is probably that the physicochemical parameter levels observed in this study might have been influenced by agricultural practices and pesticide use, which is widespread in the valley [38]. Emerging adult mosquitoes from these habitats might become more resistant towards the insecticides having the same chemical formula used in mosquito vector control [35, 39, 40].

Habitats most productive of *An. funestus* were those in higher altitude villages, which were probably less affected by agricultural insecticidal deposits [41, 42], than habitats at the floor of the valley. Nonetheless, the mosquito species from the same study villages are known to be already strongly resistant to insecticides used for public health, including pyrethroids and carbamates [43], a situation potentially related to the widespread use of pesticides in both agriculture and public health. Similar to other studies on *Anopheles* mosquitoes, the *An. funestus* habitats in this study area had weak acidity pH [40, 44]. The main habitats had pH ranging from 6.5 to 6.7, turbidity from 26.6 to 54.8 NTU and total dissolved solids from 60.5 to 80.3 mg/L, all of which are similar to most observations of habitats of *Anopheles* mosquitoes in previous studies [45, 46]. *Anopheles funestus* mosquitoes were collected from the habitats with different concentrations of nitrate, but it remains unclear whether this might influence larval development as earlier described [40].

One limitation of this study was that some characteristics such as water temperature, though included in this analysis are subject to change during the day. Future studies should consider laboratory investigations and also the use of field data collected multiple times a day to determine the suitable temperature ranges and other physicochemical characteristics for optimal survival of this mosquito species.

## Conclusion

Overall, this study has provided a basic description of *An. funestus* habitats in rural south-eastern Tanzanian districts of Ulanga and Kilombero. There were three main habitat types occupied by *An. funestus,* namely: (a) small spring-fed pools with well-defined perimeters, (b) medium-sized natural ponds retaining water most of the year, and (c) slow-moving waters along river tributaries particularly important at higher altitudes at the edge of the valley. The habitats generally had clear waters with emergent surface vegetation, depths greater than 0.5 m and distances less than 100 m from human dwellings. They were permanent or semi-permanent, retaining water most of the year. Effective control measures for this species should consider understanding their behaviour and ecology including characteristics of their aquatic habitats so that they can be targeted during their immature stages. Given the rarity of the *An. funestus* habitats and the observed characteristics, these habitats fit the description of being fixed, few and findable. Future studies should, therefore, investigate the potential of using larviciding or larval source management to improve malaria control in settings where *An. funestus* dominate.

## Data Availability

All data generated from this study will be available from the corresponding author as per request.

## Abbreviations

WHO: World Health Organization
PCR: Polymerase chain reaction
NTU: Nephelometric turbidity unit
LLINs: Long-lasting insecticide-treated nets
IRS: Indoor Residual Spraying
LC: Lower class limit
UC: Upper-class limit
